# Genetic Background of Immune Complications after Allogeneic Hematopoietic Stem Cell Transplantation in Children

**DOI:** 10.1155/2016/2626081

**Published:** 2016-01-06

**Authors:** Szymon Skoczen, Miroslaw Bik-Multanowski, Jacek J. Pietrzyk, Agnieszka Grabowska, Kamil Fijorek, Wojciech Strojny, Kinga Klus-Kwiecinska, Walentyna Balwierz, Maciej Siedlar

**Affiliations:** ^1^Department of Clinical Immunology, Chair of Clinical Immunology and Transplantation, Institute of Pediatrics, Jagiellonian University Medical College, Wielicka Street 265, 30-663 Krakow, Poland; ^2^Department of Medical Genetics, Institute of Pediatrics, Jagiellonian University Medical College, Krakow, Poland; ^3^Department of Statistics, Cracow University of Economics, Rakowicka Street 27, 31-510 Krakow, Poland; ^4^Department of Oncology and Hematology, Institute of Pediatrics, Jagiellonian University Medical College, Krakow, Poland

## Abstract

Immune reactions are among the most serious complications observed after hematopoietic stem cell transplantation (HSCT) in children. Microarray technique allows for simultaneous assessment of expression of nearly all human genes. The objective of the study was to compare the whole genome expression in children before and after HSCT. A total of 33 children referred for HSCT were enrolled in the study. In 70% of the patients HSCT was performed for the treatment of neoplasms. Blood samples were obtained before HSCT and six months after the procedure. Subsequently, the whole genome expression was assessed in leukocytes using GeneChip Human Gene 1.0 ST microarray. The analysis of genomic profiles before and after HSCT revealed altered expression of 124 genes. Pathway enrichment analysis revealed upregulation of five pathways after HSCT: allograft rejection, graft-versus-host disease, type I diabetes mellitus, autoimmune thyroid disease, and viral myocarditis. The activation of those pathways seems to be related to immune reactions commonly observed after HSCT. Our results contribute to better understanding of the genomic background of the immunologic complications of HSCT.

## 1. Introduction

Allogeneic hematopoietic stem cell transplantation (HSCT) has progressed from a risky experimental therapy to a safe and life-saving treatment modality in a relatively short span of five decades [1]. However, transplant recipients still require prolonged treatment with multiple, nonspecific, and toxic immunosuppressive drugs and are at a constant risk of immune reactions which may lead to graft-versus-host disease (GvHD) or graft rejection (GR). Immune reactions result from the activation of donor lymphocytes with subsequent recognition of the host's antigens, emergence of effector T cells, production of alloantibodies, and infiltration of tissues by alloreactive cells [2].

The aim of the study was to analyze the spectrum of alterations of genome expression resulting from HSCT.

## 2. Materials and Methods

This was a prospective study conducted from May 2009 to September 2012. The study group consisted of children and teenagers referred for HSCT, which was performed according to disease-specific treatment protocols. The study was approved by the Ethics Committee of the Jagiellonian University (KBET/96/B/2008). Written informed consent was obtained from all parents and from all patients ≥ 16 years of age.

### 2.1. Microarray Analysis

Blood samples (1.5 mL) were collected from each patient before conditioning and approximately six months after HSCT (median 6.3 months). We assessed the whole genome expression in peripheral blood leukocytes using GeneChip Human Gene 1.0 ST Array (Affymetrix, Santa Clara, USA). Total RNA extraction was performed using RiboPure Blood Kit (Ambion, Life Technologies, Carlsbad, USA). Whole transcript microarray experiment was performed according to the manufacturer's protocol (GeneChip Whole Transcript Sense Target Labeling Assay Manual, Version 4).

### 2.2. Statistical Analysis

Study sample size ensures adequate power to detect a 1.5-fold change. The microarray data were preprocessed using the R/Bioconductor package [3–5]. Robust Multiarray Average (RMA) was used for normalization [6]. Quality control was performed by investigating Principal Component Analysis (PCA), Relative Log Expression (RLE), and Normalized Unscaled Standard Error (NUSE) plots.

Moderated *t*-tests [7] were performed to detect the probes that were differentially expressed between groups, using the* limma* package [8] in the R statistical software. It was assumed that the log_2_ transformed gene expression levels are normally distributed and the between-group variation is of comparable magnitude. Multiple testing correction (Benjamini-Hochberg procedure) was applied to control the false discovery rate (FDR) [9]. Significantly different expression in the probe sets was defined as multiple comparison-corrected two-sided *p* value < 0.05.

DAVID annotation tools were used to compare gene set enrichment between the groups [10, 11]. The KEGG (Kyoto Encyclopedia of Genes and Genomes; http://www.genome.jp/kegg/) and Biocarta (http://www.biocarta.com/) pathways were selected for analysis. A set of top 250 differentially expressed genes (TOP 250 database) was exported for pathway enrichment analysis.

## 3. Results

### 3.1. Characteristic of the Study Group

The group of patients assessed before HSCT (pre-HSCT group) included 33 patients aged 1.5–19 (median 10.5) years, referred to the Stem Cell Transplantation Centre of the University Children's Hospital in Krakow. The indications for HSCT are listed in Table 1. Characteristics of children referred for HSCT are presented in Table 2.

The group of patients assessed after HSCT (post-HSCT group) included 20 children from the pre-HSCT group, aged 2.8–19.5 (median 9.6) years. In six patients in the pre-HSCT group the results of the microarray analysis were not reliable for technical reasons (poor quality of RNA sample) and another three patients were lost to follow-up. Four children died due to complications of treatment or disease progression. All patients in the post-HSCT group were treated with ablative conditioning regiments. The key clinical characteristics of the post-HSCT group are presented in Table 2.

### 3.2. Whole Genome Expression

All the primary microarray data were submitted to GEO public repository and are accessible using GEO Series accession number GSE69421 (http://www.ncbi.nlm.nih.gov/geo/query/acc.cgi?acc=GSE32472).

A summary of the differentially expressed genes is presented in Table 3.

A comparison of the pre-HSCT group and post-HSCT group revealed 124 genes with difference in expression and fold change ≥ 1.5. For 36% of these genes the expression was higher in the post-HSCT group (Table 3).

### 3.3. Pathway Enrichment Analysis

Based on the results presented in Table 4, pathway enrichment analysis was performed.

The genes showing overexpression in the post-HSCT group compared with the pre-HSCT group formed pathways responsible for donor/recipient alloaggression, including “allograft rejection” and “graft-versus-host disease,” which represent the most common complications of HSCT as well as pathways representing immunologic reaction against specific organs (“type I diabetes mellitus,” “autoimmune thyroid disease,” and “viral myocarditis”), which are among the late complications of HSCT. At the same time parallel activation of genes forming “antigen processing and presentation” pathway was observed.

The genes showing underexpression in the post-HSCT group compared with the pre-HSCT group represent pathways regulating erythropoiesis and metabolism of proteins. As analysis was performed after intensive proliferation due to reconstitution of hematopoiesis, inhibition of these genes might have occurred. The pathway “Th1/Th2 Differentiation” could be inhibited as a result of prolonged immunosuppressive treatment used for GvHD prevention in most patients treated with HSCT.

## 4. Discussion

Acute graft-versus-host disease (aGvHD) is the leading cause of morbidity and mortality after HSCT that affects the skin, liver, and gastrointestinal tract and contributes to transplant-related morbidity and mortality [2]. Approximately 50% of patients treated with HSCT subsequently develop aGvHD and require systemic treatment [12]. Chronic graft-versus-host disease (cGvHD) occurs in 40% of patients treated with HSCT from an HLA-identical sibling and in more than 50% of patients treated with HSCT from an HLA-nonidentical related donor and in 70% of patients treated with HSCT from an HLA-matched unrelated donor. The onset of symptoms is within a median of 133 days after an unrelated donor HSCT [13]. cGvHD is an immunoregulatory disorder occurring after allogeneic HSCT and has clinical features of both autoimmune disorder and immunodeficiency. The features of cGvHD resemble other autoimmune diseases such as Sjögren syndrome, scleroderma, primary biliary cirrhosis, and immune cytopenia [14]. Graft rejection (GR) occurs in 12% of children undergoing allogeneic HSCT within a median of 63 days after the transplantation [15]. Our study revealed that the genes showing overexpression in the post-HSCT group compared with the pre-HSCT group formed the pathways responsible for “allograft rejection” and “graft-versus-host disease” thus giving genetic background for the observed immune reactions. Even though no case of GR was observed in the study group, the activation of the genes responsible for GR is obvious, and our results suggest the effectiveness of the preventive treatment used in these patients.

The microarray analysis indicated presence of three other pathways: “type I diabetes mellitus,” “autoimmune thyroid disease,” and “viral myocarditis.” The incidence of compensated hypothyroidism in patients after HSCT ranges from 25 to 30% with a median latency of 2 years; overt hypothyroidism is diagnosed in up to 9% of patients with a latency of 2.7 years. Younger age is associated with higher risk of hypothyroidism [16, 17]. Patients treated with HSCT also have 2.3- to 4.0-fold higher risk of death due to cardiac causes compared with the general population [18, 19]. The cumulative incidence of cardiovascular diseases approaches 23% at 25 years after HSCT and is highest among the allogeneic HSCT recipients. The incidence appears to increase with time [20, 21]. Moreover, the patients treated with HSCT are known to have an increased risk of diabetes mellitus [22]. The risk of diabetes adjusted for age, sex, race, and BMI is 3.7 times higher in patients after allogeneic HSCT compared with the general population [23].

Activated T cells play additional important role in controlling a variety of critical steps after HSCT, such as facilitating engraftment of hematopoietic stem cells, immune reconstitution, and elimination of residual disease, but they are also responsible for immune reactions against the host's tissues [24, 25]. Our data support the hypothesis on the role of activation of gene machinery resulting in aggression of donor immune system against specific host organs leading to deterioration of their function. Regulatory T cells have been shown to mitigate immune reactions after HSCT by suppressing the early expansion of donor T cells [26].

So far, there is no validated diagnostic or predictive blood biomarker for GvHD which could improve diagnosis and prognosis and help to guide therapeutic interventions [26, 27]. Currently, the diagnosis of GvHD is based on clinical manifestations in one or more of the main target organs and on biopsy results. Moreover, once GvHD occurs, the most important predictor of long-term survival is the primary response to therapy. In patients who are resistant to initial therapy, the risk of morbidity and mortality increases significantly [28, 29]. Thus, there is a need to identify new biomarkers to predict not only GvHD development but also the survival and treatment outcomes of GvHD. Future studies might answer the question whether specific gene expression patterns can be used as biomarkers of immune complications after HSCT.

In conclusion, the results of our whole genome expression study revealed altered expression of the genes responsible for immune reactions against recipient/donor cells, proving genetic background for one of the most common complications (GvHD, GR), observed after HSCT.

## Figures and Tables

**Table 1:** The indications for HSCT (pre-HSCT group).

Condition	*n* (%)
Acute lymphoblastic leukemia (ALL)	15 (36)
Acute myeloblastic leukemia (AML)	5 (15)
Acute bilineage leukemia (ABL)	1 (3)
Juvenile myelomonocytic and acute myeloblastic leukemia (JMML/AML)	1 (3)
Myelodysplastic syndrome (MDS)	1 (3)
**Neoplasms (total)**	**23 (70)**
Severe aplastic anemia (SAA)	4 (12)
Chronic granulomatous disease (CGD)	3 (9)
Autoimmune lymphoproliferative syndrome (ALPS)	1 (3)
Hyper-IgM syndrome (HIgM)	1 (3)
Inherited neutropenia (IN)	1 (3)
**Nonneoplastic conditions (total)**	**10 (30)**

**Table 2:** Characteristics of studied groups.

Pre-HSCT group	
*N*	33
Sex	Boys 25, girls 8
Age (years)	1.5–19 (median 10.5)
Chemotherapy before HSCT (%)	23 (71%)
First line	13
Second line	9
≥third line	1
Local radiotherapy	7
Cranial (dose)	5 (12 Gy-4, 18 Gy-1)
Testes (dose)	2 (12 Gy/24 Gy, 18 Gy/18 Gy)
Time since diagnosis and patient selection (years)	Median, 1.4; range 0.08–12.9

Post-HSCT group	
*N*	20
Sex	Boys 14, girls 7
Age (years)	2.8–19.5 (median 9.6)
Conditioning regimen based on busulfan (*n*)	9 (45%)
Total body irradiation, 12 Gy/6 fractions (*n*)	6 (30%)
GvHD prophylaxis (*n*)	
Ciclosporin	2 (10%)
Methotrexate + ciclosporin	18 (90%)
GvHD (*n*)	14 (70%)
Median time from HSCT to the second assessment (range)	6.4 (6–13) months
Systemic glucocorticoids (%)	16 (80%)
Median and range of cumulative dose of glucocorticoids (equivalent of prednisone)	1463 (29–9758) mg/m^2^
Median duration of systemic glucocorticoids therapy	105 (3–240) days
Median time since discontinuation of glucocorticoids (range)	3.5 (0.4–14.3) months
Median time from discontinuation of immunosuppressive treatment to the second assessment (range) (16 patients)	1.8 (0–9) months

**Table 3:** Summary of the number of differentially expressed genes between studied groups.

Study groups	Genes with significant differences revealed after Benjamini-Hochberg correction	Number of genes with expression fold change ≥ 1.5	
		+1.5	−1.5

Post-HSCT/pre-HSCT	13 Fold change +1.5–0 −1.5–1	44	80

**Table 4:** Summary of the pathway analysis for the differentially expressed genes from TOP 250 database between the post-HSCT and pre-HSCT group.

Fold change Number of exported genes	Name of pathway map	Number of pathway maps	Published by	Participating genes (%)
Whole list 250 Underexpressed	Ribosome	hsa03010	KEGG_PATHWAY	26.5
	Porphyrin and chlorophyll metabolism	hsa00860		
	Primary immunodeficiency	hsa05340		
	Hematopoietic cell lineage	hsa05213		
	Aldosterone-regulated sodium reabsorption	hsa04960		
	Hemoglobin Chaperone	h_ahsp	BIOCARTA	10.3
	Th1/Th2 Differentiation	h_th1th2		
	3′-UTR-mediated translational regulation	REACT_1762	REACTOME_PATHWAY	17.6
	Influenza infection	REACT_6167		
	Metabolism of proteins	REACT_17015		
	Metabolism of porphyrins	REACT_9431		
	Gene expression	REACT_71		

Whole list 250 Overexpressed	Type I diabetes mellitus	hsa04940	KEGG_PATHWAY	20.8
	Allograft rejection	hsa05330		
	Graft-versus-host disease	hsa05332		
	Autoimmune thyroid disease	hsa05320		
	Viral myocarditis	hsa05416		
	Hematopoietic cell lineage	hsa05213		
	Antigen processing and presentation	hsa04612		
	Natural killer-cell mediated cytotoxicity	hsa04650		
	B-cell receptor signaling pathway	hsa04662		
	Primary immunodeficiency	hsa05340		
	Neuroactive ligand-receptor interaction	hsa04080		
	Cytokine-cytokine receptor interaction	hsa04060		
	Signaling of immune system	REACT_6900	REACTOME_PATHWAY	15.3
	CTCF	h_ctcf	BIOCARTA	8.8
	B-cell receptor complex	h_bcr		
	Inflammation mediated by chemokine and cytokine pathway	P0003	PANTHER_PATHWAY	12
	B-cell activation	P00010		

≥1.5 44	Hematopoietic cell lineage	hsa05320	KEGG_PATHWAY	19.5
	Epithelial cell signaling in *Helicobacter pylori* infection	hsa05120		
	B-cell activation	P00010	PANTHER_PATHWAY	12.2

≤−1.5 80	Porphyrin and chlorophyll metabolism	hsa00860	KEGG_PATHWAY	32.3
	Hemoglobin Chaperone	h_ahsp	BIOCARTA	16.1
	Heme biosynthesis	P02976	PANTHER_PATHWAY	14.5
	Metabolism of porphyrins	REACT_9431	REACTOME_PATHWAY	21
